# Promoting sexual and reproductive health among adolescents and young adults: an overview and equity analysis, Brazil, 2016-2025

**DOI:** 10.1590/S2237-96222026v35e20250342.en

**Published:** 2026-05-06

**Authors:** Anna Martha Vaitses Fontanari, Andréia Turmina Fontanella, Annelise Riva, João Pedro Gonçalves Krause, Júlia Colissi, Kalel Garcez, Laura dos Santos Boeira, Angelo Brandelli Costa

**Affiliations:** 1 Pontifícia Universidade Católica do Rio Grande do Sul, Porto Alegre, RS, Brazil Pontifícia Universidade Católica do Rio Grande do Sul Porto Alegre RS Brazil; 2 Universidade Federal do Rio Grande do Sul, Porto Alegre, RS, Brazil Universidade Federal do Rio Grande do Sul Porto Alegre RS Brazil; 3 Instituto Veredas, São Paulo, SP, Brazil Instituto Veredas São Paulo SP Brazil

**Keywords:** Sexual Health, Reproductive Health, Adolescent, Equity, Social Determinants of Health, Salud Sexual, Salud Reproductiva, Adolescente, Equidad, Determinantes Sociales de la Salud

## Abstract

**Objective:**

To identify sexual and reproductive health interventions aimed at promoting behavior changes and developing life skills among adolescents and young adults, considering equity criteria.

**Methods:**

An overview was conducted using searches in national and international databases (Epistemonikos, Social Systems Evidence, Health Systems Evidence, Medical Literature Analysis and Retrieval System Online - MEDLINE, Cochrane Library, and Latin American and Caribbean Health Sciences Literature - LILACS), covering publications between 2016 and 2025. Reviews addressing educational interventions in sexual and reproductive health for young people aged 10-24 years were included. The methodological quality of the studies was assessed using A Measurement Tool to Assess Systematic Reviews version 2 (AMSTAR-2), and equity analysis was conducted based on the PROGRESS framework. Key Brazilian stakeholders participated in consultative stages, contributing to the definition of the research question, interpretation of findings and discussion of barriers and facilitators to the implementation of interventions.

**Results:**

Twenty-eight systematic reviews were included. Eighteen were of critically low methodological quality, and the interventions focused mainly on the environment (n=14), the use of technologies (n=5), public policies (n=5), and facilitator or peer education (n=3).

**Conclusions:**

Comprehensive and multicomponent interventions demonstrated greater effectiveness, but there are considerable gaps in the incorporation of health equity in existing reviews.

## Resumen

**Objetivo:**

Identificar intervenciones en salud sexual y reproductiva dirigidas a promover cambios de comportamiento y desarrollar habilidades para la vida en adolescentes y jóvenes, considerando criterios de equidad.

**Métodos:**

Se realizó overview mediante búsquedas en bases de datos nacionales e internacionales (Epistemonikos, Social Systems Evidence, Health Systems Evidence, Medical Literature Analysis and Retrieval System Online - Medline, Cochrane Library y Literatura Latinoamericana y del Caribe en Ciencias de la Salud - Lilacs), abarcando publicaciones entre 2016 y 2025. Se incluyeron revisiones que abordaron intervenciones educativas en salud sexual y reproductiva para jóvenes de 10 a 24 años. La calidad metodológica de los estudios se evaluó utilizando la herramienta A Measurement Tool to Assess Systematic Reviews versión 2 (AMSTAR-2), y el análisis de equidad se realizó con base en el marco PROGRESS. Actores brasileños clave participaron en las etapas consultivas, contribuyendo a la definición de la pregunta de investigación, la interpretación de los hallazgos y el análisis de las barreras y los facilitadores para la implementación de las intervenciones.

**Resultados:**

Se incluyeron veintiocho revisiones sistemáticas. Dieciocho presentaron una calidad metodológica críticamente baja, y las intervenciones se centraron principalmente en el entorno (n=14), el uso de tecnologías (n=5), las políticas públicas (n=5) y la educación por facilitadores o pares (n=3).

**Conclusiones:**

Las intervenciones integrales y multicomponentes demostraron mayor efectividad, pero existen importantes deficiencias en la incorporación de la equidad en salud en las revisiones existentes.

Ethical aspects

This research used public domain anonymized databases.

## Introduction

The social determinants of health suggest inequities and encompass social, economic and environmental factors that affect well-being ^1^. Ignoring inequalities can compromise the effectiveness of interventions in systematic reviews ^2^. People in poverty or stigmatized situations benefit more from interventions that consider their needs ^3^^,^^4^, and the Equity-Effectiveness Loop theoretical framework indicates that interventions can be up to two-thirds less effective in vulnerable groups if they do not receive customized attention ^5^. Socioeconomic status, barriers to access to health, inaccurate diagnoses and adherence challenges impact the effectiveness of health actions ^5^^,^^6^^,^^7^.

Sexual health can be defined as a state of well-being related to one's own sexuality; this state includes physical, emotional, mental and social perceptions ^8^^,^^9^, based on a positive and respectful approach that ensures pleasurable and safe sexual experiences, without coercion, discrimination or violence, and that promotes reproductive rights and reproductive health ^10^.

Health behaviors are formed early in life, and interventions focused on the sexual health of young people can influence knowledge, attitudes and self-efficacy ^11^^,^^12^. Furthermore, interventions improve communication, encourage safe practices, increase the use of health services and reduce adverse outcomes such as sexually transmitted infections (STIs) and unintended pregnancies ^11^^,^^12^. Interventions are heterogeneous and vary in form from online participation ^13^; mass media ^14^; through to sex education in schools or universities ^15^. They promote essential knowledge, skills and values ​​^16^^,^^17^, encourage healthy relationships and shape perceptions ^18^^,^^19^, as well as promoting well-being and preventing sexual violence ^8^^,^^20^^,^^21^.

Comprehensive interventions have promoted knowledge and behavior changes ^24^, while technological interventions have promoted knowledge acquisition and reduction of risky behaviors, in addition to involving parents in promoting safer sex ^22^. Multicomponent approaches have been effective in promoting adolescent sexual and reproductive health and preventing bullying ^23^. On the other hand, abstinence programs have been ineffective. However, none of these previous overviews considered health equity when interpreting their findings.

In the field of sexual and reproductive health, for example, Black women face higher rates of physical and sexual victimization during adolescence ^25^^,^^26^^,^^27^. Even with fewer partners and consistent condom use, they face higher rates of infection by the human immunodeficiency virus (HIV) and other STIs compared to White and Hispanic women ^25^^,^^28^. Sociocultural factors, such as racial and gender discrimination, lead to underreporting of violence ^29^^,^^30^^,^^31^.

This overview aimed to identify sexual and reproductive health interventions focused on promoting behavior changes and developing life skills among young people in different contexts, considering evidence of effectiveness and equity criteria.

## Methods

Design

This is an overview, conducted in accordance with the guidelines of the Preferred Reporting Items for Systematic Reviews and Meta-Analyses and those of the Cochrane Handbook for Systematic Reviews of Interventions ^32^^,^^42^. The study included engagement steps with key Brazilian stakeholders, in accordance with national guidelines for social participation in health ^33^.

Key stakeholders were involved in multiple phases of the study, participating in an advisory capacity but without co-authorship of the manuscript. Research groups, policymakers and representatives of civil society, including non-governmental organizations (such as Reprolatina), were invited via email. Selection was based on criteria of institutional diversity, experience in public policy and work in sexual and reproductive health.

Twelve representatives from universities, civil society organizations, health professionals and public service managers participated. These stakeholders contributed to defining the guiding concepts and the research question, aiming to reflect the challenges faced in practice. Finally, they reviewed a summary of the evidence and participated in a deliberative dialogue, in which barriers and facilitators to the implementation of the interventions identified were discussed.

Key stakeholder engagement took place through focus groups. The process was documented by means of recording and transcription. The information obtained was used for thematic categorization and content analysis to refine the research question and contextualize the final synthesis.

Eligibility criteria

The studies included in the overview were necessarily systematic reviews or overviews (due to the possibility of capturing systematic reviews that could have been included as references in those works). The inclusion criteria for studies were: (i) gathering information from participants aged 10-24 years, as per the World Health Organization definition of youth and young adults ^34^; (ii) addressing sexual and reproductive education interventions; (iii) being a peer-reviewed indexed article; (iv) being published in English, Spanish or Portuguese; and (v) being published between 2016 and 2025.

The publication time frame was adopted in order to examine reviews published after the launch of the Global Strategy for Women's, Children's and Adolescents' Health (2016-2030) and the Global Accelerated Action for the Health of Adolescents, which represent a theoretical and political milestone in adolescent sexual and reproductive health. There were no restrictions based on the location where the systematic reviews were conducted or the studies included in them.

In this overview, the following key concepts were adopted. 

Sex education interventions: actions planned to promote the sexual health and autonomy of individuals, changing an existing situation or addressing a particular problem ^35^. They can occur at individual or community levels, with collaboration from multiple sectors and key stakeholders. In sexual and reproductive health, they can aim to prevent consequences such as unintended pregnancy, STI and HIV infection ^36^. 

Behavior changes: alterations in risky behaviors, such as unprotected sex, and adoption of safe sex practices, such as consistent condom use.

Life skills: psychosocial skills that enable young people to make informed decisions and face challenges, such as communication skills and self-efficacy ^37^.

Social determinants of health: social, economic, environmental and cultural factors that influence the health and well-being of individuals ^1^.

We excluded reviews that: had not been peer-reviewed (grey literature, such as theses, dissertations and posters, that had not undergone a formal review process); were not systematic reviews or overviews (e.g., primary studies); did not include results on the effectiveness of interventions; and were not available in full text.

Information sources

The search was conducted on August 30, 2022, and updated on May 19, 2025, in the following databases: Epistemonikos, Social Systems Evidence, Health Systems Evidence, Medline (via PubMed), Cochrane Library, and Latin American and Caribbean Health Sciences Literature (LILACS) via the Virtual Health Library. References from relevant publications, suggestions made by key stakeholders and sources such as books and conference proceedings were selected to identify potentially eligible additional studies (overviews or systematic reviews). Citation lists of all previously published included articles were manually inspected; key stakeholders for relevant references were contacted; and other sources were searched manually, such as reference lists of relevant books and conference proceedings.

Search strategy

A pilot strategy was developed for the Medline database (via PubMed) and adapted for other databases, using controlled descriptors from the Medical Subject Headings vocabulary. The final detailed strategy for bibliographic databases is shown in Table 1. A filter was applied for both systematic reviews and publication date, but no filter was applied for country of publication. Studies published in English, Spanish and Portuguese were included. The searches were conducted by two authors (LSB and AMVF) and validated by a third (ABC). 

Selection process

Study screening was performed by pairs of independent authors (AMVF and JC, AR and JPGK, ATF and KG), with conflict resolution by a third reviewer (LSB) using Microsoft Excel. The excluded studies are shown in (Supplementary Table 1).

Data collection process

The references were extracted using Mendeley software, enabling removal of duplicates. They were then imported to an Excel spreadsheet. The inclusion and exclusion criteria were applied independently by two reviewers (AMVF and LSB). In case of conflict, a third reviewer was consulted (ABC).

Data list

Two researchers (LSB and AMVF) independently extracted data from the studies using an electronic spreadsheet (Excel). Disagreements were resolved by consensus or by a third reviewer (ABC). Information was extracted on: type of intervention (school intervention, public policy, technology-based), target population (adolescents, young adults), outcomes assessed (use of contraceptives, STIs, unintended pregnancy), quantitative results (measures of association, proportions), study location (low/middle/high income countries), methodological quality of the reviews (AMSTAR-2), and inclusion of equity-related aspects (PROGRESS).

**Table 1:** Search strategy used for each source. Brazil, 2016-2025

Database	Search strategy (2016-2025)
BVS^a^/Lilacs^b^	("sex education" OR "educação sexual" OR "educación sexual") AND (adolescent OR teenagers OR "young adults" OR youth OR adolescentes OR "jovens adultos" OR jovens OR joven OR jóvenes) AND (type_of_study:("systematic_reviews" OR "policy_brief" OR "overview")) AND (year_cluster:[2016 TO 2025]) OU ("life skills" AND adolescent AND (sex OR sexual) AND (type_of_study:("systematic_reviews" OR "policy_brief" OR "overview")) AND (year_cluster:[2016 TO 2025]))
Cochrane Library	("sex education" OR "educação sexual" OR "educación sexual") AND (adolescent OR teenagers OR "young adults" OR youth OR adolescentes OR "jovens adultos" OR jovens OR Joven OR Jóvenes) in Title Abstract Keyword OU ("life skills" AND adolescent AND (sex or sexual) in Title Abstract Keyword)
Epistemonikos	("sex education" OR "educação sexual" OR "educación sexual") AND (adolescent OR teenagers OR "young adults" OR youth OR adolescentes OR "jovens adultos" OR jovens OR Joven OR Jóvenes) OU ("life skills" AND adolescent AND (sex or sexual)) Filtro: Article type: Systematic review; Publication date: Custom range: 2016-01-01 to 2025-05-19
Health Systems Evidence	sex education OU "life skills" AND adolescent AND (sex or sexual) Filtro: Year: 2016-2025
Medline^c^ (via PubMed)	("sex education" OR "educação sexual" OR "educación sexual") AND (adolescent OR teenagers OR "young adults" OR youth OR adolescentes OR "jovens adultos" OR jovens OR Joven OR Jóvenes) OU ("life skills" AND adolescent AND (sex or sexual)) Filtro: Article type: Systematic review; Publication date: Custom range: 2016-01-01 to 2025-05-19
Social Systems Evidence	sex education OU "life skills" AND adolescent AND (sex or sexual) Filtro: Year: 2016-2025

a BVS: Biblioteca Virtual em Saúde (Virtual Health Library); bLilacs: Literatura Latino-Americana e do Caribe em Ciências da Saúde (Latin American and Caribbean Health Sciences Literature); cMedline: Medical Literature Analysis and Retrieval System Online.

Assessment of risk of bias in the studies 

Assessment of methodological quality and risk of bias was conducted by two independent reviewers (LSB and AMVF) using A Measurement Tool to Assess Systematic Reviews 2 (AMSTAR-2) ^38^, which consists of 16 items. Each item was classified as "yes", "no" or "partially yes", according to the degree of conformity with the methodological criteria. The scores were subsequently grouped and categorized into levels of methodological confidence: high (0-1 non-critical item), moderate (>1 non-critical item), low (1 critical item) or critically low (>1 critical item).

Considerations about equity

The PROGRESS tool ^39^ was used for equity assessment. PROGRESS is an acronym for place of residence, race/ethnicity (including language and culture), occupation, gender/sex, religion, education, socioeconomic status and social capital ^40^. The framework facilitates a systematic approach to understanding the causes of health inequities ^41^.

In this study, the PROGRESS domains were used to identify potentially disadvantaged population subgroups and to assess whether the interventions included in the systematic reviews considered equity aspects in their design, implementation or outcome analysis. Information was extracted on the presence or absence of data disaggregated by these factors, as well as on specific strategies aimed at reducing inequalities.

Synthesis methods

A narrative synthesis of the evidence was made, with thematic categorization and grouping of findings by type of intervention (school, technological, public policy), age group, and implementation context. The synthesis was complemented by a deliberative dialogue with key stakeholders, who discussed barriers and facilitators to the implementation of the interventions identified.

## Results

Initial screening resulted in identification of 264 publications in the databases, of which 263 were assessed by title and abstract after removing duplicates. Twenty-eight systematic reviews were included in the narrative synthesis of the findings (Figure 1; Table 2).

Of the 28 studies included, 18 were classified as having critically low methodological quality; 6 as low; 3 as moderate; and 1 as high. The following stand out among the main problems observed in the critically low quality reviews: absence of a registered study protocol, lack of a complete list of excluded studies, and absence of risk of bias assessment of the primary studies included (Table 2). One review was classified as high quality, as it presented a registered protocol, clear methodological criteria and systematic risk of bias assessment.

Although some reviews included data on social determinants, there were gaps in the analysis of the effect of these factors on the outcomes of the interventions (Table 3). A lack of interventions focused on racial and cultural equity and gender diversity was also noted.

Most studies focused on outcomes such as contraceptive use (n=16), unintended pregnancy rates (n=9), STIs (n=6), and changes in knowledge about sexual health (n=13). Interventions occurred mainly in school settings (n=13), public policies (n=5) or were technology-based (n=5). Comprehensive and multicomponent interventions, especially those based in schools and on technology, were effective in promoting sexual and reproductive health among young people (Table 2). Based on the AMSTAR-2 assessment, most studies were of critically low (n=15) or low (n=7) quality ^14^^,^^19^^,^^43^^,^^64^^,^^65^, and 3 were of moderate quality ^22^^,^^66^^,^^67^.

**Figure 1:**
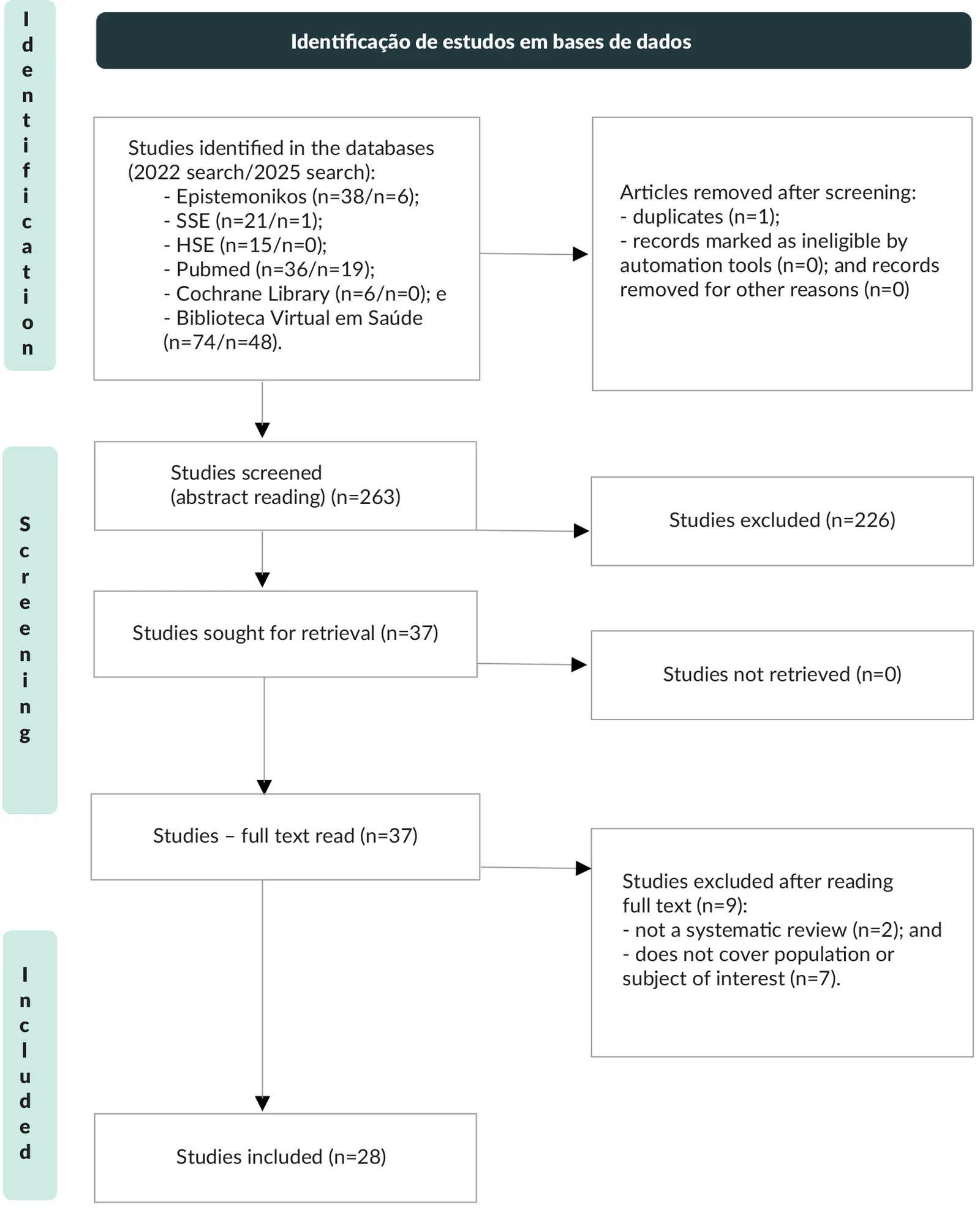
Process for selection and inclusion of studies in the review. Brazil, 2016-2025 (n=28)

**Table 2:** Main characteristics of the studies included in the review. Brazil, 2016-2025 (n=28)

Author, year	Place	Population (age)	Setting	Outcome observed	AMSTAR-2
Aguilar-Quesada, 2024 ^67^	Global	Adolescents (10-19 years)	Technology-based (school)	Knowledge, attitudes, use of contraceptives	Moderate
Aslan, 2022 ^49^	Turkey	Students (elementary education/high school/university)	School	Knowledge, intentions/behaviors	Critically low
Denford, 2016 ^24^	Global	Adolescence	School	Risky sexual behavior, pregnancy, HIV^b^	Critically low
Dodd, 2022 ^66^	Global	Adolescents (11-18 years)	Peer education (school)	Knowledge, attitudes, risk behaviors	Moderate
Garzón-Orjuela, 2020 ^22^	Global	Adolescence (13-18 years)	School	Pregnancy, STI^c^, risky sexual behavior	Moderate
Goldfarb, 2021 ^8^	Global	Children and adolescents (school)	School	Violence prevention, healthy relationships	Critically low
González, 2025 ^65^	Latin America	Adolescents (10-19 years)	Technology-based (sites/chatbots)	Use of contraceptives, knowledge, attitudes	Low
Hindin, 2016 ^56^	L/M-income countries	Youth (10-24 years)	Public policies	Unintended/repeat pregnancy, use of contraceptives	Low
Jouriles, 2017 ^51^	Global	Young adults (at least 18 years)	University (sexual violence)	Prosocial attitudes and beliefs	Critically low
Lameiras-Fernández, 2021 ^9^	High-income countries	Adolescents	School	Sexual behaviors, condom use	Critically low
Lopez, 2016 ^20^	United States, United Kingdom, Mexico, South Africa	Participants (10-25 years)	School	Use of contraceptives, pregnancy, STI/HIV	High
Lopez, 2016 ^20^	United States, United Kingdom, Mexico, South Africa	Participants (10-25 years)	Health services/clinics	Use of contraceptives and condoms, pregnancy	High
Malhotra, 2021 ^62^	L/M-income countries	School age	Public policy	Child marriage	Low
Martin, 2020 ^13^	Global	Youth (10-24 years)	Technology-based (on-line)	Sexual behaviors, knowledge	Critically low
Mason-Jones, 2016 ^47^	Global	Adolescents (10-19 years)	School	HIV, STI, pregnancy, condom use	High
Mirzazadeh, 2017 ^19^	Global (Focus on United States)	Students (1^st^ grade to high school)	School	STI/HIV, sexual initiation, condom use	High
Mwale, 2018 ^63^	Sub-Saharan Africa	Adolescents (under 24 years)	School	Knowledge, attitudes, condom use, STI/pregnancy	Critically low
Marseille, 2018 ^46^	United States and Canada	School age	School	Pregnancy, sexual initiation, condom use	Low
Oringanje ^59^	Global	Adolescents	Public policy	Unintended pregnancy, STI/HIV, contraception	High
Orozco-Olvera, 2019 ^14^	Global	Adolescents	Technology-based (mass media)	Knowledge, attitudes, sexual behaviors	High
Peterson, 2019 ^48^	Global	Adolescents (10-19 years)	School (environment)	Pregnancy, sexual initiation, contraception use	Low
Ribas, 2021 ^21^	Latin America and Caribbean	Adolescents	Public policies	Unintended pregnancy, behaviors	Critically low
Sani, 2016 ^57^	Sub-Saharan Africa	Adolescents (school)	School	Condom use, STI/HIV	High
Shackleton, 2016 ^23^	Global	Students (11-18 years)	School (bullying/violence)	Sexual health, bullying, pregnancy	Critically low
Siddiqui, 2020 ^15^	India	Adolescents (10-24 years)	Peer education	Knowledge, attitudes, behaviors	Critically low
Sun, 2018 ^58^	Developed countries	Adolescents (under 24 years)	Peer education	Knowledge, attitudes, behaviors	Critically low
Torres-Cortés, 2023 ^64^	Global	Adolescents (10-19 years)	Education with facilitators	Knowledge, attitudes, behaviors	Low
Widman, 2018 ^43^	United States	Adolescents (14-18 years)	Technology-based	Condom use, abstinence	High

a AMSTAR-2: A Measurement Tool to Assess Systematic Reviews version 2; bHIV: human immunodeficiency virus; cSTI: sexually transmitted infections.

**Table 3:** Main characteristics relating to equity (PROGRESS). Brazil, 2016-2025 (n=28)

Progress	Barriers to access to intervention	Strategies to mitigate barriers to access to intervention
Place of residence	- Certain places of residence (such as rural areas) may be far from services where ongoing sex education activities take place. - Barriers to improving sexual and reproductive health in low- and middle-income countries can be very different from those in more developed countries.	- Education through entertainment is a possibility to promote positive sexual behaviors, especially due to its wide coverage in rural areas and in low- and middle-income countries ^14^. - Online interventions, especially when they contain participatory components, can facilitate access to sexual and reproductive health ^13^^,^^14^^,^^43^. - Decentralizing sexual and reproductive health activities, promoting community-based engagement, as well as public policies (e.g., conditional cash transfers to maintain access to education and health) are more effective. Such strategies have proven particularly effective in promoting sexual and reproductive health in low- and middle-income countries ^21^^,^^56^.
Race/ethnicity/ culture/ language	- Curriculum-based sexual and reproductive education programs are less effective at reaching young Black people. - Societies in which sexuality is considered taboo may present challenges to the implementation of sexual and reproductive health interventions.	- Considering contextual factors when developing comprehensive sexual and reproductive education strategies is essential ^22^^,^^24^^,^^59^. For example, taking active steps to include Black youth, incorporating representative images into educational materials and creating media outlets that celebrate racial diversity. - Strategies that focus exclusively on sexual health education in schools may be ineffective in the face of structural and economic barriers ^48^.
Occupation	- Lack of job opportunities and youth unemployment are linked to high rates of teenage pregnancy.	- Having a favorable labor market for women, or at least a visible promise of economic opportunities, is capable of delaying the age of marriage among girls ^62^. - Macro policies that support female employment are capable of affecting a large number of young women in low- and middle-income countries ^62^.
Gender/sex	- Girls are more affected by child marriage, unintended or unwanted pregnancies, and sexual abuse. - Some interventions have been effective in changing the sexual behavior of young men, but not that of young women.	- Interventions focused on student development, which encouraged girls' school attendance and progression through cash transfers, have been more successful in preventing child marriage. In contrast, targeted interventions, even those aimed at female empowerment, did not show effectiveness in preventing child marriage. - In some contexts, gender-specific interventions may be more effective than gender-neutral interventions ^47^^,^^51^^,^^63^. - Teaching topics based on social justice, human rights and equality tools can increase knowledge, awareness and appreciation of gender equity and sexual rights ^8^.
Religion	- In areas with conservative sexual and reproductive education policies, maintaining sexual and reproductive education in the academic curriculum can be particularly complex.	- Providing information about contraception in schools, communities and media channels accessed by adolescents is fundamental to diversifying sources of reliable information. - Faith-based interventions can have an impact on reducing unintended or unwanted pregnancies among young people. However, practices focused solely on abstinence may increase risky behaviors.
Education	- People who cannot read or have low reading skills may have difficulty with written materials (booklets, pamphlets).	- Develop audiovisual materials and other tools to facilitate interaction with information on sexual and reproductive education. - Conduct periodic assessments of sexual health needs and offer simpler, more cost-effective strategies, such as posters, which appear to be effective among young adults currently enrolled in higher education.
Socioeconomic status	- Demonstrations of condom use and other skills-based activities are hampered in classrooms with a lot of students. - Scarcity of computers makes it difficult to engage in technology-based activities.	- Income transfer policies conditioned on maintaining access to education and health are capable of preventing unintended or unwanted pregnancies among adolescents.
Social capital	- People with disabilities are not taken into consideration in sexual health interventions. - The possibility of housing instability is disregarded in sexual health interventions.	- Map misconceptions and offer training for educators to develop methodologies sensitive to people with disabilities. Include people with disabilities in general sexual and reproductive education classes, offer learning support such as sign language interpretation/audio description, and develop curricula specifically designed to address the main myths related to the sexuality of these adolescents. - Establish partnerships with non-governmental organizations that offer specific support for young people in situations of housing instability.

We identified three groups of interventions, either in-person or online, conducted by trained professionals or between peers: interventions in schools, health centers and universities. Sexual and reproductive health promotion policies were predominantly implemented in schools ^44^^,^^45^^,^^46^^,^^64^. Comprehensive sexual health interventions and inclusive approaches were more effective in promoting sexual diversity, preventing intimate partner violence, developing healthy relationships, preventing child sexual abuse, encouraging social/emotional learning and promoting preventive sexual behaviors ^8^^,^^64^. Most studies highlighted the importance of a multidisciplinary approach ^8^^,^^19^^,^^20^^,^^23^^,^^47^^,^^48^. One systematic review also highlighted the relevance of facilitator training for developing skills to implement the intervention ^64^.

Three reviews were conducted to assess the effectiveness of the impact of online interventions on factors such as condom use, sexually transmitted infections, unintended pregnancies and perceptions of sexual health ^13^^,^^14^^,^^43^. These included games or quizzes providing information, participatory components, communities that enabled discussions between participants, educational sessions and personalized assistance. The evidence showed that online interventions positively impacted condom use and abstinence, and their broad coverage made them effective strategies for developing populations.

High-quality evidence has indicated that online interventions have positively impacted condom use and abstinence, regardless of demographic factors or program format ^14^^,^^43^. Television, radio, video and comic book interventions on safe sex practices have promoted a reduction in unprotected sex and an increase in STI testing ^14^. Due to its wide reach, education through entertainment has emerged as an effective strategy for promoting positive sexual behaviors, especially in developing countries ^14^.

Schools have proven to be suitable for providing sexual and reproductive health services. There is little evidence that curricular educational programs alone, without provision of contraceptives and condoms, are effective in reducing risky behaviors among adolescents and improving their health outcomes 8,9,19,20,22,23,46,47,48,49.

Brief educational interventions that take place in primary care services and family planning clinics were more effective than traditional counseling methods in promoting the use of contraceptives and condoms among young people ^20^. These interventions comprised 1 to 3 sessions, lasting 15 to 60 minutes each, conducted individually and in groups. The methods ranged from direct oral communication to use of audiovisual tools.

Over the years, interventions have been developed to reduce sexual violence on university campuses ^50^^,^^51^. These have included self-defense training programs, interventions focused on bystanders and distribution of educational posters. Although school and university environments are key locations for implementing sex education programs ^24^, use of the internet has been able to contribute to sexual and reproductive health education programs, offering ease of access, availability, low cost and remote or asynchronous participation ^52^^,^^53^^,^^54^. Among adolescents, social media platforms have proven to be the main source of information about sexuality ^55^.

## Discussion

This is the first overview to analyze evidence on sexual and reproductive health interventions focusing on health equity. Multicomponent interventions, especially those based in schools, have proven effective. These findings confirmed that comprehensive, rgente n nente interventions are effective in promoting sexual and reproductive health among rgen people. Such interventions demonstrated a greater rgente n knowledge and reduction of risky behaviors compared to approaches that promote rge abstinence, which were ineffective.

This rgente analysis demonstrated that, although 28 reviews were included in the qualitative synthesis, 18 presented critically low methodological quality, raising concerns about the reliability of the available evidence in the field. The most frequent interventions occurred in school settings (n=13), but those based on public policies (n=5) and technology (n=5) were also relevant. Although some systematic reviews included characterization of participants based on social determinants of health, none of them analyzed the effect of the interventions from na equity perspective.

By applying the PROGRESS framework, we identified that factors such as race, gender, socioeconomic status and social capital affect effectiveness and can amplify inequalities. A gap was found in interventions focused on racial and cultural equity and gender diversity. Curriculum-based programs may be less effective in addressing Black rgen, potentially exacerbating inequalities ^22^^,^^24^^,^^59^^,^^60^.

Sex education often lacks teaching methods geared toward this population. In order to mitigate this disparity, it is essential to promote inclusion, using representative materials and media that celebrate racial diversity ^61^. In the gender domain, absence of effective strategies for sexual and reproductive health has disproportionately negative consequences for girls, such as child marriage, unintended or unwanted pregnancies and sexual abuse. Structural interventions, such as those that support girls’ school attendance and progress through conditional cash transfers, have proven more successful in preventing child marriage compared to actions focused solely on female empowerment ^62^.

The high rate of rgen unemployment rgente a determining rgen in maintaining high rates of teenage pregnancy ^21^. Technology-based interventions positively impact condom use and abstinence ^19^, and their wide coverage ^14^ makes them viable strategies for populations in developing contexts and rural rgen. Mass media interventions (TV, radio) have promoted a reduction in unprotected sex and na increase in STI testing ^14^.

The need to address health equity is crucial. Ignoring inequalities can rgente n n the effectiveness of interventions in systematic reviews ^2^. In the domain of socioeconomic status, income transfer policies conditioned on maintaining access to education and health have been able to prevent unintended or unwanted pregnancies among rgente n ne ^21^, benefiting poor women more. The combination of sex education, contraception and rgente n engagement has reduced unintended pregnancies among rgen people aged 10-24 in low- and rgent-income countries ^56^.

In the school rgente , curricular educational programs alone, without rgente n ne contraceptives and condoms, are not very effective in reducing risky behaviors in rgente n ne 8,9,19,20,22,23,46,47,48,49. A rgente n nente approach, which combines education with health services ^20^ and involves rgente /caregivers ^43^, is more effective in promoting safe sex and reducing risky behaviors. Integrating sex education with a framework of social justice and human rights can increase knowledge and appreciation of gender equality and sexual rights ^8^^,^^64^.

Cultural resistance rgente a significant barrier ^49^, and implementation of sex education interventions must consider the sociocultural rgente . The failure of interventions to overcome structural and economic barriers to the promotion of sexual and reproductive health has been documented ^48^. In short, although comprehensive and rgente n nente interventions are the main recommendation, the findings of this overview emphasize the rgente need for future reviews and primary studies to not rge include social determinants, but also to actively analyze their differential effect on the effectiveness of interventions.

This review had three main limitations: exclusion of grey literature (theses and dissertations) due to the criterion of absence of peer review; the fact that the included systematic reviews provided few details about the interventions (duration, topics and training of the professionals involved); and failure to analyze duplication of primary studies in the reviews, heterogeneity of studies or publication bias. The exclusion criterion of the article not being available for download may have restricted the collection of evidence, and is considered another limitation of this study.

This overview was the first to apply na equity lens to assessment of the effectiveness of sexual and reproductive health interventions targeting rgente n ne and rgen adults. The results confirmed that comprehensive, rgente n nente interventions, especially those based in schools and using technology, were the most effective in promoting sexual and reproductive health, demonstrating a greater rgente n knowledge and reduction of risky behaviors compared to programs focused solely on abstinence. The equity analysis revealed a crucial gap: while the interventions consider social determinants of health, most systematic reviews have failed to analyze the differential effect of these factors on outcomes.

This finding suggests that factors such as race, gender, socioeconomic status and social capital affect the effectiveness of interventions and can amplify inequalities. Thus, this synthesis points to the rgente need for future research and public policies to actively consider and analyze these determinants to ensure more inclusive interventions that are sensitive to social inequalities.

## Data Availability

All data used for this overview, including the extraction matrix and the assessment of the quality of the studies, are available by contacting the corresponding author: angelo.costa@pucrs.br
