# Supplementary material for: Promoting sexual and reproductive health among adolescents and young adults: an overview and equity analysis, Brazil, 2016-2025
Source: Epidemiol Serv Saude. 2026 May 6;35:e20250342. doi: 10.1590/S2237-96222026v35e20250342.en (PMC13686304; doi:10.1590/S2237-96222026v35e20250342.en)
Supplement: Supplementary file 1 [file 2237-9622-ress-35-e20250342-Suplementar-tabela1.pdf]

Tabela Suplementar 1. Motivo da exclusão das publicações avaliadas em texto completo. Brasil, 2016-2025 (n=9)

Motivo de exclusão	Referências
Não é uma revisão sistemática (segundo critérios Cochrane)	Goldfarb ES, Lieberman LD. Three decades of research: the case for comprehensive sex education. J Adolesc Health. 2021;68(1):13-27.
	Pound P, Denford S, Shucksmith J, Tanton C, Johnson AM, Owen J, et al. What is best practice in sex and relationship education? A synthesis of evidence, including stakeholders' views. BMJ Open. 2017;7(5):e014791.
Não aborda população ou tema de interesse	Berendes D, Smith J, Berendes J, Cullen K, Han E, Shrestha P, et al. Sexual health interventions delivered to participants by mobile technology: a systematic review and meta-analysis of randomised controlled trials. PLoS Med. 2021;18(11):e1003752.
	Brunton G, Dickson K, Caird J, Stokes G, Thomas J. Sexual health promotion and contraceptive services in local authorities: a systematic review of economic evaluations 2010-2015. Health Technol Assess. 2016;20(20):1-162.
	Burrus BB, Krieger K, Rutledge R, Rabre A, Axelson S, Miller A, et al. Building bridges to a brighter tomorrow: a systematic evidence review of interventions that prepare adolescents for adulthood. Am J Public Health. 2018;108(Suppl 1):S25-31.
	Flowers P, Riddell J, Park C, Ahmed B, Young I, Frankis J, et al. The clinical effectiveness of individual behaviour change interventions to reduce risky sexual behaviour after a negative HIV test in men who have sex with men: systematic and realist reviews and intervention development. Health Technol Assess. 2017;21(5):1-164.
	Kang H. Indigenous women and youth in the sex trade: a systematic review of culturally relevant support systems for exiting the trade. J Ethn Subst Abuse. 2019;18(3):405-27.
	McCann E, Marsh L, Brown M. People with intellectual disabilities, relationship and sex education programmes: a systematic review. J Intellect Disabil. 2019;23(4):434-49.
	Moncrieff G, Finlayson K, Cordey S, McCrimmon R, Harris C, Barreix M, et al. First and second trimester ultrasound in pregnancy: a systematic review and metasynthesis of the views and experiences of pregnant women, partners, and health workers. PLoS One. 2021;16(12):e0261096.
