# Supplementary material for: Promoting sexual and reproductive health among adolescents and young adults: an overview and equity analysis, Brazil, 2016-2025
Source: Epidemiol Serv Saude. 2026 May 6;35:e20250342. doi: 10.1590/S2237-96222026v35e20250342.en (PMC13686304; doi:10.1590/S2237-96222026v35e20250342.en)
Supplement: Supplementary file 2 [file 2237-9622-ress-35-e20250342-Supplementary-table1.pdf]

Supplementary Table 1. Reason for excluding full-text assessed publications. Brazil, 2016-2025 (n=9)

Reason for exclusion	References
Not a systematic review (according to Cochrane criteria)	Goldfarb ES, Lieberman LD. Three decades of research: the case for comprehensive sex education. J Adolesc Health. 2021;68(1):13-27.
	Pound P, Denford S, Shucksmith J, Tanton C, Johnson AM, Owen J, et al. What is best practice in sex and relationship education? A synthesis of evidence, including stakeholders' views. BMJ Open. 2017;7(5):e014791.
Does not cover population or subject of interest	Berendes D, Smith J, Berendes J, Cullen K, Han E, Shrestha P, et al. Sexual health interventions delivered to participants by mobile technology: a systematic review and meta-analysis of randomised controlled trials. PLoS Med. 2021;18(11):e1003752.
	Brunton G, Dickson K, Caird J, Stokes G, Thomas J. Sexual health promotion and contraceptive services in local authorities: a systematic review of economic evaluations 2010-2015. Health Technol Assess. 2016;20(20):1-162.
	Burrus BB, Krieger K, Rutledge R, Rabre A, Axelson S, Miller A, et al. Building bridges to a brighter tomorrow: a systematic evidence review of interventions that prepare adolescents for adulthood. Am J Public Health. 2018;108(Suppl 1):S25-31.
	Flowers P, Riddell J, Park C, Ahmed B, Young I, Frankis J, et al. The clinical effectiveness of individual behaviour change interventions to reduce risky sexual behaviour after a negative HIV test in men who have sex with men: systematic and realist reviews and intervention development. Health Technol Assess. 2017;21(5):1-164.
	Kang H. Indigenous women and youth in the sex trade: a systematic review of culturally relevant support systems for exiting the trade. J Ethn Subst Abuse. 2019;18(3):405-27.
	McCann E, Marsh L, Brown M. People with intellectual disabilities, relationship and sex education programmes: a systematic review. J Intellect Disabil. 2019;23(4):434-49.
	Moncrieff G, Finlayson K, Cordey S, McCrimmon R, Harris C, Barreix M, et al. First and second trimester ultrasound in pregnancy: a systematic review and metasynthesis of the views and experiences of pregnant women, partners, and health workers. PLoS One. 2021;16(12):e0261096.
