# Supplementary material for: Promoting sexual and reproductive health among adolescents and young adults: an overview and equity analysis, Brazil, 2016-2025
Source: Epidemiol Serv Saude. 2026 May 6;35:e20250342. doi: 10.1590/S2237-96222026v35e20250342.en (PMC13686304; doi:10.1590/S2237-96222026v35e20250342.en)
Supplement: Translation

# Translation: Promoção da saúde sexual e reprodutiva entre adolescentes e jovens adultos: overview e análise de equidade, Brasil, 2016-2025

**DOI:** 10.1590/S2237-96222026v35e20250342.pt

**Authors:** Anna Martha Vaitses Fontanari, Andréia Turmina Fontanella, Annelise Riva, João Pedro Gonçalves Krause, Júlia Colissi, Kalel Garcez, Laura dos Santos Boeira, Angelo Brandelli Costa

**Affiliations:** 1 Pontifícia Universidade Católica do Rio Grande do Sul, Porto Alegre, RS, Brasil Pontifícia Universidade Católica do Rio Grande do Sul Porto Alegre RS Brasil; 2 Universidade Federal do Rio Grande do Sul, Porto Alegre, RS, Brasil Universidade Federal do Rio Grande do Sul Porto Alegre RS Brasil; 3 Instituto Veredas, São Paulo, SP, Brasil Instituto Veredas São Paulo SP Brasil

**Keywords:** Saúde Sexual, Saúde Reprodutiva, Adolescente, Equidade, Determinantes Sociais da Saúde

## Abstract

**Objetivo:**

Identificar intervenções em saúde sexual e reprodutiva voltadas à promoção de mudanças comportamentais e ao desenvolvimento de habilidades para a vida entre adolescentes e jovens adultos, considerando critérios de equidade.

**Métodos:**

Realizou-se overview com buscas em bases nacionais e internacionais (Epistemonikos, Social Systems Evidence, Health Systems Evidence, Medical Literature Analysis and Retrieval System Online - Medline, Biblioteca Cochrane e Literatura Latino-Americana e do Caribe em Ciências da Saúde - Lilacs), abrangendo publicações entre 2016 e 2025. Foram incluídas revisões que abordavam intervenções educativas em saúde sexual e reprodutiva para jovens de 10-24 anos. A qualidade metodológica dos estudos foi avaliada por meio da ferramenta A Measurement Tool to Assess Systematic Reviews versão 2 (AMSTAR-2), e a análise de equidade foi conduzida com base na estrutura Progress. Atores-chave brasileiros participaram de etapas consultivas, contribuindo para a definição da pergunta de pesquisa, interpretação dos achados e discussão sobre barreiras e facilitadores à implementação das intervenções.

**Resultados:**

Foram incluídas 28 revisões sistemáticas. Dezoito eram de qualidade metodológica criticamente baixa, e as intervenções focaram principalmente no ambiente escolar (n=14), no uso de tecnologias (n=5), em políticas públicas (n=5) e na educação de facilitadores ou pares (n=3).

**Conclusões:**

Intervenções abrangentes e multicomponentes demonstraram maior efetividade, mas há lacunas consideráveis na incorporação da equidade em saúde nas revisões existentes.

Aspectos éticos

A pesquisa utilizou banco de dados de domínio público e anonimizados.

## Introdução

Os determinantes sociais da saúde sugerem iniquidades e englobam fatores sociais, econômicos e ambientais que afetam o bem-estar ^1^. Ignorar desigualdades pode comprometer a eficácia das intervenções em revisões sistemáticas ^2^. Pessoas em situação de pobreza ou estigmatizadas são mais beneficiadas por intervenções que consideram suas necessidades ^3^^,^^4^, e o modelo teórico do Ciclo de Eficácia de Equidade indica que intervenções podem ser até dois terços menos eficazes em grupos vulneráveis caso não recebam atenção customizada ^5^. O status socioeconômico, as barreiras ao acesso à saúde, os diagnósticos imprecisos e os desafios de adesão impactam a eficácia das ações de saúde ^5^^,^^6^^,^^7^.

A saúde sexual pode ser definida como um estado de bem-estar relacionado à própria sexualidade; esse estado inclui percepções físicas, emocionais, mentais e sociais ^8^^,^^9^, baseando-se em uma abordagem positiva e respeitosa que garante experiências sexuais prazerosas e seguras, sem coerção, discriminação ou violência, e que promove direitos e saúde reprodutiva ^10^.

Comportamentos de saúde se formam cedo na vida, e intervenções voltadas à saúde sexual dos jovens podem influenciar o conhecimento, as atitudes e a autoeficácia ^11^^,^^12^. Ainda, melhoram a comunicação, incentivam práticas seguras, aumentam o uso de serviços de saúde e reduzem desfechos adversos, como infecções sexualmente transmissíveis (IST) e gestações indesejadas ^11^^,^^12^. As intervenções se apresentam de forma heterogênea e variam na forma de participação on-line ^13^; de mídia de massa ^14^; até educação sexual em escolas ou em universidades ^15^. Promovem conhecimento, habilidades e valores essenciais ^16^^,^^17^, incentivam relações saudáveis e moldam percepções ^18^^,^^19^, além de promoverem o bem-estar e prevenirem violência sexual ^8^^,^^20^^,^^21^.

Intervenções abrangentes promoveram conhecimento e mudanças comportamentais ^24^, ao passo que intervenções tecnológicas promoveram a aquisição de conhecimento e a redução de comportamentos de risco, além de terem envolvido os pais na promoção do sexo protegido ^22^. Já abordagens multicomponentes foram eficazes na promoção da saúde sexual e reprodutiva adolescente e na prevenção de bullying ^23^. Por outro lado, programas de abstinência foram ineficazes. No entanto, nenhum dos overviews anteriores considerou a equidade em saúde ao interpretar os achados.

No campo da saúde sexual e reprodutiva, por exemplo, mulheres negras enfrentam maiores taxas de vitimização física e sexual na adolescência ^25^^,^^26^^,^^27^. Mesmo apresentando menos parceiros e usando preservativos consistentemente, enfrentam taxas elevadas de vírus da imunodeficiência humana (HIV) e outras IST em comparação com mulheres brancas e hispânicas ^25^^,^^28^. Fatores socioculturais, como discriminação racial e de gênero, levam à subnotificação de violência ^29^^,^^30^^,^^31^. 

Este overview objetivou identificar intervenções em saúde sexual e reprodutiva voltadas à promoção de mudanças comportamentais e ao desenvolvimento de habilidades de vida entre jovens em diferentes contextos, considerando evidências de eficácia e critérios de equidade.

## Métodos

Delineamento do estudo

Trata-se de overview, conduzido conforme as diretrizes do Preferred Reporting Items for Systematic Reviews and Meta-Analyses e do Cochrane Handbook for Systematic Reviews of Interventions ^32^^,^^42^. O estudo incluiu etapas de engajamento com atores-chave brasileiros, conforme diretrizes nacionais de participação social em saúde ^33^. 

Atores-chave foram envolvidos em múltiplas fases do estudo, com participação consultiva e sem coautoria no manuscrito. Foram convidados, por e-mail, grupos de pesquisa, formuladores de políticas e representantes da sociedade civil, incluindo organizações não governamentais (como a Reprolatina). A seleção considerou critérios de diversidade institucional, experiência em políticas públicas e atuação em saúde sexual e reprodutiva. 

Participaram 12 representantes de universidades, organizações da sociedade civil, profissionais da saúde e gestores públicos. Esses atores contribuíram para a definição dos conceitos-guia e da pergunta de pesquisa, com o objetivo de refletir os desafios enfrentados na prática. Ao final, revisaram um resumo das evidências e participaram de diálogo deliberativo, no qual foram discutidas barreiras e facilitadores para a implementação das intervenções identificadas. 

O engajamento dos atores-chave se deu por meio de grupos focais. O registro foi realizado através de gravação e transcrição. As informações obtidas foram utilizadas para categorização temática e análise de conteúdo para aprimorar a pergunta de pesquisa e contextualizar a síntese final.

Critérios de elegibilidade

Os estudos incluídos eram necessariamente revisões sistemáticas ou overviews (pela possibilidade de captar revisões sistemáticas que poderiam ter sido incluídas como referências nesses trabalhos). Os critérios de inclusão de estudos foram: (i) reunir informações de participantes de 10-24 anos, conforme definição de jovens e jovens adultos da Organização Mundial de Saúde ^34^; (ii) abordar intervenções de educação sexual e reprodutiva; (iii) ser um artigo revisado por pares indexado; (iv) ser publicado em inglês, espanhol ou português; e (v) ser publicado entre 2016 e 2025. 

O recorte temporal foi adotado para observar revisões publicadas a partir do lançamento da Global Strategy for Women's, Children's and Adolescents' Health (2016-2030) e da Ação Global Acelerada para a Saúde de Adolescentes que representam um marco teórico-político em saúde sexual e reprodutiva de adolescentes. Não houve restrição por local de realização das revisões sistemáticas ou dos estudos incluídos nelas.

Neste overview, os conceitos-chave, a seguir, foram adotados. 

Intervenções de educação sexual: ações planejadas para promover a saúde e a autonomia sexual dos indivíduos, mudando uma situação existente ou abordando um problema particular ^35^. Elas podem ocorrer em níveis individuais ou comunitários, com colaboração de múltiplos setores e atores-chave. Na saúde sexual e reprodutiva, podem visar prevenir consequências como gestação indesejada, IST e infecção pelo HIV ^36^. 

Mudanças comportamentais: alterações em comportamentos de risco, como sexo desprotegido, e adoção de práticas sexuais seguras, como o uso consistente de preservativos.

Habilidades para a vida: competências psicossociais que capacitam os jovens a tomar decisões informadas e a enfrentar desafios, como a capacidade de comunicação e a autoeficácia ^37^.

Determinantes sociais da saúde: fatores sociais, econômicos, ambientais e culturais que influenciam a saúde e o bem-estar dos indivíduos ^1^.

Foram excluídas revisões que não: apresentavam revisão por pares (literatura cinzenta, como teses, dissertações e pôsteres, que não passaram por processo de revisão formal); eram sistemáticas ou overviews (por exemplo, estudos primários); incluíam resultados de efetividade de intervenções; e estavam disponíveis em texto completo.

Fontes de informação

A busca foi realizada em 30 de agosto de 2022 e atualizada em 19 de maio de 2025, nas seguintes bases de dados: Epistemonikos, Social Systems Evidence, Health Systems Evidence, Medline (via PubMed), Biblioteca Cochrane e Literatura Latino-Americana e do Caribe em Ciências da Saúde (Lilacs) via Biblioteca Virtual em Saúde. Foram selecionadas as referências de publicações relevantes, sugestões de atores-chave e fontes como livros e anais de conferências científicas para identificar estudos adicionais potencialmente elegíveis (overviews ou revisões sistemáticas). As listas de citações de todos os artigos incluídos previamente publicados foram inspecionadas manualmente; atores-chave para referências relevantes foram contatados; e outras fontes foram buscadas manualmente, como listas de referências de livros relevantes e anais de conferências.

Estratégia de busca

Uma estratégia-piloto foi desenvolvida para a base de dados Medline (via PubMed) e adaptada para as demais bases de dados, utilizando os descritores controlados do vocabulário Medical Subject Headings. A estratégia final detalhada para as bases de dados bibliográficos foi apresentada na Tabela 1. Aplicou-se um filtro tanto para revisões sistemáticas quanto para data de publicação, mas não foi aplicado filtro para país de publicação. Foram incluídos estudos publicados em inglês, espanhol e português. As buscas foram realizadas por dois autores (LSB e AMVF) e validadas por um terceiro (ABC). 

Processo de seleção

A triagem dos estudos foi realizada por duplas de autores independentes (AMVF e JC, AR e JPGK, ATF e KG), com resolução de conflitos por um terceiro revisor (LSB) utilizando Microsoft Excel. Os estudos excluídos foram registrados na (Tabela Suplementar 1.)

Processo de coleta de dados

As referências foram extraídas para o software Mendeley, permitindo a remoção de duplicatas; depois, foram carregadas em uma planilha do Excel. Foram aplicados por dois revisores (AMVF e LSB), independentemente, os critérios de inclusão e exclusão. Em caso de conflito, um terceiro revisor era consultado (ABC).

Lista de dados

Dois pesquisadores (LSB e AMVF) extraíram independentemente os dados dos estudos por meio de planilha eletrônica (Excel). Divergências foram resolvidas por consenso ou por um terceiro revisor (ABC). Foram extraídas informações sobre: tipo de intervenção (intervenção escolar, política pública, baseada em tecnologia), população-alvo (adolescentes, jovens adultos), desfechos avaliados (uso de contraceptivos, IST, gravidez indesejada), resultados quantitativos (medidas de associação, proporções), local de realização dos estudos (países de baixa/média/alta renda), qualidade metodológica das revisões (AMSTAR-2) e inclusão de aspectos relacionados à equidade (Progress).

**Tabela 1:** Estratégia de busca utilizada em cada fonte. Brasil, 2016-2025

Banco de dados	Estratégia de busca (2016-2025)
BVS^a^/Lilacs^b^	("sex education" OR "educação sexual" OR "educación sexual") AND (adolescent OR teenagers OR "young adults" OR youth OR adolescentes OR "jovens adultos" OR jovens OR joven OR jóvenes) AND (type_of_study:("systematic_reviews" OR "policy_brief" OR "overview")) AND (year_cluster:[2016 TO 2025]) OU ("life skills" AND adolescent AND (sex OR sexual) AND (type_of_study:("systematic_reviews" OR "policy_brief" OR "overview")) AND (year_cluster:[2016 TO 2025]))
Cochrane Library	("sex education" OR "educação sexual" OR "educación sexual") AND (adolescent OR teenagers OR "young adults" OR youth OR adolescentes OR "jovens adultos" OR jovens OR Joven OR Jóvenes) in Title Abstract Keyword OU ("life skills" AND adolescent AND (sex or sexual) in Title Abstract Keyword)
Epistemonikos	("sex education" OR "educação sexual" OR "educación sexual") AND (adolescent OR teenagers OR "young adults" OR youth OR adolescentes OR "jovens adultos" OR jovens OR Joven OR Jóvenes) OU ("life skills" AND adolescent AND (sex or sexual)) Filtro: Article type: Systematic review; Publication date: Custom range: 2016-01-01 to 2025-05-19
Health Systems Evidence	sex education OU "life skills" AND adolescent AND (sex or sexual) Filtro: Year: 2016-2025
Medline^c^ (via PubMed)	("sex education" OR "educação sexual" OR "educación sexual") AND (adolescent OR teenagers OR "young adults" OR youth OR adolescentes OR "jovens adultos" OR jovens OR Joven OR Jóvenes) OU ("life skills" AND adolescent AND (sex or sexual)) Filtro: Article type: Systematic review; Publication date: Custom range: 2016-01-01 to 2025-05-19
Social Systems Evidence	sex education OU "life skills" AND adolescent AND (sex or sexual) Filtro: Year: 2016-2025

a BVS: Biblioteca Virtual em Saúde; ^b^Lilacs: Literatura Latino-Americana e do Caribe em Ciências da Saúde; ^c^Medline: Medical Literature Analysis and Retrieval System Online

Avaliação do risco de viés dos estudos

A avaliação da qualidade metodológica e do risco de viés foi conduzida por dois revisores independentes (LSB e AMVF) por meio da ferramenta A MeaSurement Tool to Assess Systematic Reviews 2 (AMSTAR-2) ^38^, composta por 16 itens. Cada item foi classificado como "sim", "não" ou "parcialmente sim", conforme o grau de conformidade com os critérios metodológicos. Os escores foram posteriormente agrupados e categorizados em níveis de confiança metodológica: alto (0-1 item não crítico), moderado (>1 item não crítico), baixo (1 item crítico) ou criticamente baixo (>1 item crítico).

Considerações sobre equidade

Foi empregada a ferramenta Progress ^39^ para avaliação de equidade. Progress é um acrônimo para local de residência, raça (incluindo língua e cultura), ocupação, gênero, religião, educação, status socioeconômico e capital social ^40^. A estrutura facilita uma abordagem sistemática para entender as causas das iniquidades em saúde ^41^. 

Neste estudo, os domínios do Progress foram utilizados para identificar subgrupos populacionais potencialmente desfavorecidos e avaliar se as intervenções incluídas nas revisões sistemáticas consideraram aspectos de equidade em sua concepção, implementação ou análise de resultados. Foram extraídas informações sobre a presença ou ausência de dados desagregados por esses fatores, bem como sobre estratégias específicas voltadas à redução de desigualdades.

Métodos de síntese

Foi realizada síntese narrativa das evidências, com categorização temática e agrupamento dos achados por tipo de intervenção (escolar, tecnológica, política pública), faixa etária e contexto de implementação. A síntese foi complementada por diálogo deliberativo com atores-chave, que discutiram barreiras e facilitadores à implementação das intervenções identificadas. 

## Resultados

A triagem inicial resultou na identificação de 264 publicações nas bases de dados, das quais 263 foram avaliadas por título e resumo após a remoção de duplicatas. Vinte e oito revisões sistemáticas foram incluídas na síntese narrativa dos achados (Figura 1; Tabela 2).

Dos 28 estudos incluídos, 18 foram classificados como de qualidade metodológica criticamente baixa; 6, como de baixa; 3, como de moderada; e 1, como de alta. Entre os principais problemas observados nas revisões de qualidade criticamente baixa, destacam-se: ausência de protocolo de estudo registrado, falta de lista completa de estudos excluídos e ausência de avaliação do risco de viés dos estudos primários incluídos (Tabela 2). Uma revisão foi classificada como de alta qualidade, por apresentar protocolo registrado, critérios metodológicos claros e avaliação sistemática do risco de viés. 

Embora algumas revisões tenham incluído dados de determinantes sociais, há uma lacunas na análise do efeito desses fatores nos resultados das intervenções (Tabela 3). Notou-se, também, um vazio de intervenções focadas em equidade racial e cultural e diversidade de gênero. 

A maioria dos estudos focou desfechos como uso de anticoncepcional (n=16), taxas de gestação indesejada (n=9), IST (n=6) e mudanças em conhecimentos sobre saúde sexual (n=13). As intervenções ocorreram principalmente em ambientes escolares (n=13), políticas públicas (n=5) e com base tecnológica (n=5). Intervenções abrangentes e multicomponentes, especialmente as baseadas em escolas e tecnologias, foram eficazes na promoção da saúde sexual e reprodutiva entre jovens (Tabela 2). Com base na avaliação AMSTAR-2, a maioria dos estudos foi de qualidade criticamente baixa (n=15) ou baixa (n=7) ^14^^,^^19^^,^^43^^,^^64^^,^^65^, e 3 foram de qualidade moderada ^22^^,^^66^^,^^67^.

**Figura 1:** Processo de seleção e inclusão dos estudos na revisão. Brasil, 2016-2025 (n=28)

**Tabela 2:** Principais características dos estudos incluídos na revisão. Brasil, 2016-2025 (n=28)

Autor, ano	Local	População (idade)	Contexto	Desfecho observado	AMSTAR-2^a^
Aguilar-Quesada, 2024 ^67^	Global	Adolescentes (10-19 anos)	Baseada em tecnologia (escolar)	Conhecimentos, atitudes, uso de contraceptivos	Moderada
Aslan, 2022 ^49^	Turquia	Estudantes (ensino fundamental/médio/universitário)	Escolar	Conhecimento, intenções/comportamentos	Criticamente baixa
Denford, 2016 ^24^	Global	Adolescência	Escolar	Comportamento sexual de risco, gravidez, HIV^b^	Criticamente baixa
Dodd, 2022 ^66^	Global	Adolescentes (11-18 anos)	Educação entre pares (escolar)	Conhecimentos, atitudes, comportamentos de risco	Moderada
Garzón-Orjuela, 2020 ^22^	Global	Adolescência (13-18 anos)	Escolar	Gravidez, IST^c^, comportamento sexual de risco	Moderada
Goldfarb, 2021 ^8^	Global	Crianças e adolescentes (escolar)	Escolar	Prevenção da violência, relações saudáveis	Criticamente baixa
González, 2025 ^65^	América Latina	Adolescentes (10-19 anos)	Baseada em tecnologia (sites/chatbots)	Uso de contraceptivos, conhecimentos, atitudes	Baixa
Hindin, 2016 ^56^	Países de B/M Renda	Jovens (10-24 anos)	Políticas públicas	Gravidez não planejada/repetida, uso de contraceptivos	Baixa
Jouriles, 2017 ^51^	Global	Jovens adultos (mínimo: 18 anos)	Universitário (violência sexual)	Atitudes e crenças pró-sociais	Criticamente baixa
Lameiras-Fernández, 2021 ^9^	Países de alta renda	Adolescentes	Escolar	Comportamentos sexuais, uso de preservativos	Criticamente baixa
Lopez, 2016 ^20^	Estados Unidos, Reino Unido, México, África do Sul	Participantes (10-25 anos)	Escolar	Uso de contraceptivos, gravidez, IST/HIV	Alta
Lopez, 2016 ^20^	Estados Unidos, Reino Unido, México, África do Sul	Participantes (10-25 anos)	Serviços de saúde/clínicas	Uso de contraceptivos e preservativos, gravidez	Alta
Malhotra, 2021 ^62^	Países de B/M Renda	Idade escolar	Política pública	Casamento infantil	Baixa
Martin, 2020 ^13^	Global	Jovens (10-24 anos)	Baseada em tecnologia (on-line)	Comportamentos sexuais, conhecimento	Criticamente baixa
Mason-Jones, 2016 ^47^	Global	Adolescentes (10-19 anos)	Escolar	HIV, IST, gravidez, uso de preservativo	Alta
Mirzazadeh, 2017 ^19^	Global (Foco Estados Unidos)	Alunos (1º ao ensino médio)	Escolar	IST/HIV, iniciação sexual, uso de preservativos	Alta
Mwale, 2018 ^63^	África Subsaariana	Adolescentes (menores 24 anos)	Escolar	Conhecimento, atitudes, uso de preservativos, IST/gravidez	Criticamente baixa
Marseille, 2018 ^46^	Estados Unidos e Canadá	Idade escolar	Escolar	Gravidez, iniciação sexual, uso de preservativos	Baixa
Oringanje ^59^	Global	Adolescentes	Política Pública	Gravidez indesejada, IST/HIV, contracepção	Alta
Orozco-Olvera, 2019 ^14^	Global	Adolescentes	Baseada em tecnologia (mídia de massa)	Conhecimento, atitudes, comportamentos sexuais	Alta
Peterson, 2019 ^48^	Global	Adolescentes (10-19 anos)	Escolar (ambiente)	Gravidez, iniciação sexual, uso de contracepção	Baixa
Ribas, 2021 ^21^	América Latina e Caribe	Adolescentes	Políticas públicas	Gravidez indesejada, comportamentos	Criticamente baixa
Sani, 2016 ^57^	África Subsaariana	Adolescentes (escolar)	Escolar	Uso de preservativo, IST/HIV	Alta
Shackleton, 2016 ^23^	Global	Estudantes (11-18 anos)	Escolar (bullying/violência)	Saúde sexual, bullying, gravidez	Criticamente baixa
Siddiqui, 2020 ^15^	Índia	Adolescentes (10-24 anos)	Educação entre pares	Conhecimentos, atitudes, comportamentos	Criticamente baixa
Sun, 2018 ^58^	Países desenvolvidos	Adolescentes (menores 24 anos)	Educação entre pares	Conhecimentos, atitudes, comportamentos	Criticamente baixa
Torres-Cortés, 2023 ^64^	Global	Adolescentes (10-19 anos)	Educação com facilitadores	Conhecimentos, atitudes, comportamentos	Baixa
Widman, 2018 ^43^	Estados Unidos	Adolescentes (14-18 anos)	Baseada em tecnologia	Uso de preservativo, abstinência	Alta

a AMSTAR-2: A Measurement Tool to Assess Systematic Reviews versão 2; bHIV: vírus da imunodeficiência humana; cIST: infecções sexualmente transmissíveis.

**Tabela 3:** Principais características referentes à equidade (Progress). Brasil, 2016-2025 (n=28)

Progress	Barreiras de acesso à intervenção	Estratégias para mitigar as barreiras de acesso à intervenção
Local de residência	- Certos locais de residência (como áreas rurais) podem estar distantes dos serviços onde ocorrem atividades contínuas de educação sexual. - Barreiras para melhorar a saúde sexual e reprodutiva em países de baixa e média renda podem ser muito diferentes dos países mais desenvolvidos.	- Educação por entretenimento é uma possibilidade para promover comportamentos sexuais positivos, especialmente pela ampla cobertura em áreas rurais e em países de baixa e média renda ^14^. - Intervenções on-line, especialmente quando contêm componentes participativos, podem facilitar o acesso à saúde sexual e reprodutiva ^13^^,^^14^^,^^43^. - Descentralizar as atividades de saúde sexual e reprodutiva, promovendo o engajamento baseado na comunidade, bem como políticas públicas (por exemplo, transferências condicionais de dinheiro para manter o acesso à educação e à saúde) são mais eficazes. Tais estratégias mostraram-se particularmente eficazes na promoção da saúde sexual e reprodutiva em países de baixa e média renda ^21^^,^^56^.
Raça, etnia, cultura, língua	- Programas de educação sexual e reprodutiva baseados no currículo são menos eficazes em alcançar jovens negros. - Sociedades onde a sexualidade é considerada um tabu podem apresentar desafios à implementação de intervenções sobre saúde sexual e reprodutiva.	- Considerar fatores contextuais ao desenvolver estratégias abrangentes de educação sexual e reprodutiva é essencial ^22^^,^^24^^,^^59^. Por exemplo, adotar esforços ativos para incluir a juventude negra, incorporando imagens representativas em materiais educativos e criando meios de comunicação que celebrem a diversidade racial. - Estratégias que visam exclusivamente à educação para a saúde sexual em escolas podem ser inócuas diante das barreiras estruturais e econômicas ^48^.
Ocupação	- A falta de oportunidades de emprego e o desemprego juvenil estão relacionados às altas taxas de gestação entre adolescentes.	- Ter um mercado de trabalho favorável para as mulheres ou, pelo menos, uma promessa visível de oportunidades econômicas, é capaz de adiar a idade do casamento entre as meninas ^62^. - Políticas macro que apoiam o emprego feminino são capazes de afetar um grande número de jovens mulheres de países de baixa e média renda ^62^.
Gênero	- Meninas são mais afetadas por casamento infantil, gestações não planejadas ou indesejadas e abuso sexual. - Algumas intervenções foram eficazes em mudar o comportamento sexual de jovens homens, mas não o de jovens mulheres.	- Intervenções no desenvolvimento estudantil, que incentivaram a presença das meninas na escola e a progressão por meio de transferências em dinheiro, obtiveram maior sucesso na prevenção do casamento infantil. Em contraste, intervenções direcionadas, mesmo quando voltadas para o empoderamento feminino, não mostraram eficácia na prevenção do casamento infantil. - Em alguns contextos, intervenções específicas de gênero podem ser mais eficazes do que intervenções neutras em relação ao gênero ^47^^,^^51^^,^^63^. - Tópicos de ensino baseados em ferramentas de justiça social, direitos humanos e igualdade podem aumentar o conhecimento, a conscientização e o apreço pela equidade de gênero e pelos direitos sexuais ^8^.
Religião	- Em áreas com políticas conservadoras de educação sexual e reprodutiva, pode ser particularmente complexo manter a educação sexual e reprodutiva no currículo acadêmico.	- Oferecer informações sobre contracepção em escolas, comunidades e canais de mídia acessados por adolescentes é fundamental para diversificar as fontes de informação confiável. - Intervenções baseadas na fé podem ter um impacto na redução de gestações não planejadas ou indesejadas entre os jovens. No entanto, práticas focadas apenas na abstinência podem aumentar comportamentos de risco.
Educação	- Pessoas que não sabem ler ou têm baixa capacidade de leitura podem ter dificuldades com materiais escritos (livretos, folhetos).	- Desenvolver materiais audiovisuais e outros facilitadores de interação com informações sobre educação sexual e reprodutiva. - Realizar diagnóstico periódico sobre as demandas de saúde sexual e oferecer estratégias mais simples e econômicas, como cartazes, que parecem ser eficazes entre jovens adultos com ensino superior em andamento.
Status socioeconômico	- Demonstrações de uso de preservativos e outras atividades baseadas em habilidades têm a implementação prejudicada em turmas com muitos alunos. - A escassez de computadores dificulta a prática de atividades baseadas em tecnologia.	- Políticas de transferência de renda condicionadas à manutenção do acesso à educação e à saúde são capazes de prevenir gestações não planejadas ou indesejadas entre adolescentes.
Capital social	- Pessoas com deficiência não são consideradas em intervenções de saúde sexual. - A possibilidade de instabilidade habitacional é desconsiderada em intervenções de saúde sexual.	- Mapear as concepções e oferecer treinamento para educadores desenvolverem metodologias sensíveis a pessoas com deficiência. Incluir pessoas com deficiência em aulas gerais de educação sexual e reprodutiva, oferecer suporte para aprendizado como tradução para Libras/ audiodescrição e elaborar currículos especialmente projetados para abordar os principais mitos relacionados à sexualidade desses adolescentes. - Estabelecer parcerias com organizações não governamentais que oferecem suporte específico para jovens em situação de instabilidade habitacional.

Foram identificados 3 grupos de intervenções, presenciais ou on-line, realizadas por profissionais treinados ou entre pares: intervenções em escolas, unidades de saúde e universidades. As políticas de promoção de saúde sexual e reprodutiva apresentaram predominante implementação nas escolas ^44^^,^^45^^,^^46^^,^^64^. Intervenções abrangentes de saúde sexual e abordagens inclusivas foram mais eficazes em promover a diversidade sexual, prevenir violência entre parceiros íntimos, desenvolver relacionamentos saudáveis, prevenir abuso sexual infantil, incentivar aprendizagem social/emocional e promover comportamentos sexuais preventivos ^8^^,^^64^. A maioria dos estudos destacou a importância da abordagem multidisciplinar ^8^^,^^19^^,^^20^^,^^23^^,^^47^^,^^48^. Uma revisão sistemática destacou, ainda, a relevância do treinamento dos facilitadores para o desenvolvimento de habilidades para implementar a intervenção ^64^.

Três revisões foram conduzidas para avaliar a eficácia do impacto das intervenções on-line em fatores como o uso de preservativos, infecções sexualmente transmissíveis, gestações indesejadas e percepções sobre saúde sexual de intervenções on-line ^13^^,^^14^^,^^43^. Estas incluíram jogos ou *quizzes* fornecendo informações, componentes participativos, comunidades que permitiram discussões entre participantes, sessões educacionais e assistência personalizada. As evidências mostraram que as intervenções on-line impactaram positivamente o uso de preservativos e a abstinência, e sua ampla cobertura as tornaram estratégias eficazes para populações em desenvolvimento.

Evidências de alta qualidade indicaram que intervenções on-line impactaram positivamente o uso de preservativos e a abstinência, independentemente de fatores demográficos ou do formato do programa ^14^^,^^43^. Intervenções de televisão, rádio, vídeos e quadrinhos sobre práticas sexuais seguras promoveram redução do sexo desprotegido e aumento da testagem de IST ^14^. Devido à sua ampla cobertura, a educação via entretenimento surgiu como estratégia eficaz para promover comportamentos sexuais positivos, especialmente em países em desenvolvimento ^14^.

As escolas demonstraram ser adequadas para a prestação de serviços de saúde sexual e reprodutiva. Existem poucas evidências de que os programas educacionais curriculares sozinhos e sem o fornecimento de co ntraceptivos e preservativos sejam eficazes na redução de comportamentos de risco em adolescentes e na melhoria dos seus resultados de saúde 8,9,19,20,22,23,46,47,48,49.

Intervenções educativas breves que ocorrem em serviços de atenção primária e clínicas de planejamento familiar foram mais eficazes do que os métodos tradicionais de aconselhamento na promoção do uso de contraceptivos e preservativos entre os jovens ^20^. Essas intervenções compreenderam de 1 a 3 sessões, com duração de 15 a 60 minutos cada, realizadas individualmente e em grupo. Os métodos variaram de comunicação oral direta até o uso de ferramentas audiovisuais.

Ao longo dos anos, intervenções foram desenvolvidas para reduzir a violência sexual nas universidades ^50^^,^^51^. Destacaram-se programas de treinamento de autodefesa, intervenções focadas em transeuntes e distribuição de cartazes educativos. Embora os ambientes escolar e universitário sejam locais-chave para a implementação de programas de educação sexual ^24^, o uso da internet pôde contribuir para programas de educação em saúde sexual e reprodutiva, apresentando facilidade de acesso, disponibilidade, baixo custo e participação remota ou assíncrona ^52^^,^^53^^,^^54^. Entre os adolescentes, as plataformas de redes sociais demonstraram ser a principal fonte de obtenção de informações sobre sexualidade ^55^.

## Discussão

Este é o primeiro overview a analisar as evidências sobre intervenções de saúde sexual e reprodutiva com foco na equidade em saúde. As intervenções multicomponentes, especialmente aquelas de base escolar, mostraram-se eficazes. Estes achados confirmaram que intervenções abrangentes e multicomponentes são eficazes na promoção da saúde sexual e reprodutiva entre jovens. Tais intervenções demonstraram maior impacto no conhecimento e na redução de comportamentos de risco em comparação com abordagens que promovem apenas a abstinência, as quais foram ineficazes.

Esta análise demonstrou que, embora 28 revisões tenham sido incluídas na síntese qualitativa, 18 apresentaram qualidade metodológica criticamente baixa, o que levanta preocupações sobre a confiabilidade das evidências disponíveis na área. As intervenções mais frequentes ocorreram em ambientes escolares (n=13), mas as baseadas em políticas públicas (n=5) e em tecnologia (n=5) também foram relevantes. Apesar de algumas revisões sistemáticas terem incluído a caracterização dos participantes com base em determinantes sociais da saúde, nenhuma delas analisou o efeito das intervenções sob a ótica da equidade. 

Ao aplicar a estrutura Progress, identificou-se que fatores como raça, gênero, status socioeconômico e capital social afetam a eficácia e podem ampliar as iniquidades. Constatou-se uma lacuna em intervenções focadas em equidade racial e cultural e diversidade de gênero. Programas baseados no currículo podem ser menos eficazes na abordagem de jovens negros, potencialmente agravando as desigualdades ^22^^,^^24^^,^^59^^,^^60^. 

A educação sexual frequentemente carece de instrução voltada para essa população. Para mitigar essa disparidade, é essencial promover a inclusão, utilizando materiais representativos e mídias que celebrem a diversidade racial ^61^. No domínio de gênero, a ausência de estratégias eficazes para a saúde sexual e reprodutiva tem consequências desproporcionalmente negativas para as meninas, como casamento infantil, gestações não planejadas ou indesejadas e abuso sexual. Intervenções estruturais, como as que apoiam a frequência escolar e o progresso das meninas por meio de transferências de renda condicionadas, mostraram-se mais bem-sucedidas na prevenção do casamento infantil em comparação com ações focadas apenas no empoderamento feminino ^62^. 

O alto índice de desemprego juvenil também é um fator determinante na manutenção das altas taxas de gestação na adolescência ^21^. Intervenções baseadas em tecnologia impactam positivamente o uso de preservativos e a abstinência ^19^, e sua ampla cobertura ^14^ as torna estratégias viáveis para populações em contextos de desenvolvimento e áreas rurais. Intervenções de mídia de massa (TV, rádio) promoveram redução do sexo desprotegido e aumento da testagem de IST ^14^.

A necessidade de abordar a equidade em saúde é crucial. Ignorar desigualdades pode comprometer a eficácia das intervenções em revisões sistemáticas ^2^. No domínio de status socioeconômico, as políticas de transferência de renda condicionadas à manutenção do acesso à educação e à saúde foram capazes de prevenir gestações não planejadas ou indesejadas entre adolescentes ^21^, beneficiando mais mulheres pobres. A combinação de educação sexual, contracepção e engajamento comunitário reduziu gestações indesejadas junto a jovens de 10-24 anos em países de baixa e média renda ^56^. 

No contexto escolar, os programas educacionais curriculares isoladamente, sem fornecimento de contraceptivos e preservativos, são pouco eficazes na redução de comportamentos de risco em adolescentes 8,9,19,20,22,23,46,47,48,49. A abordagem multidisciplinar, que combina educação com serviços de saúde ^20^ e envolve pais/cuidadores ^43^, é mais eficaz para promover sexo protegido e reduzir comportamentos de risco. A integração da educação sexual com um arcabouço de justiça social e direitos humanos pode aumentar o conhecimento e a valorização da equidade de gênero e dos direitos sexuais ^8^^,^^64^. 

A resistência cultural também é uma barreira significativa ^49^, e a implementação de intervenções em educação sexual deve considerar o contexto sociocultural. A falha das intervenções em superar barreiras estruturais e econômicas à promoção de saúde sexual e reprodutiva foi documentada ^48^. Em suma, embora as intervenções abrangentes e multicomponentes sejam a principal recomendação, os achados deste overview enfatizam a necessidade urgente de que futuras revisões e estudos primários não apenas incluam determinantes sociais, mas que ativamente analisem o seu efeito diferenciado na eficácia das intervenções.

Esta revisão apresentou três limitações principais: a exclusão da literatura cinzenta (teses e dissertações), devido ao critério de ausência de revisão por pares; o fato de as revisões sistemáticas incluídas terem fornecido poucos detalhes sobre as intervenções (duração, tópicos e formação dos profissionais envolvidos); e a não realização de análise de duplicidade de estudos primários nas revisões, heterogeneidade dos estudos ou viés de publicação. O critério de exclusão de não ter o artigo disponível para download pode ter restringido a captação de evidências, sendo considerada outra limitação do estudo. 

Este overview foi o primeiro a aplicar uma lente de equidade na avaliação da eficácia de intervenções de saúde sexual e reprodutiva destinadas a adolescentes e jovens adultos. Os resultados confirmaram que intervenções abrangentes e multicomponentes, especialmente aquelas baseadas em escolas e tecnologias, foram as mais eficazes na promoção da saúde sexual e reprodutiva, demonstrando maior impacto no conhecimento e na redução de comportamentos de risco em comparação com programas focados apenas na abstinência. A análise de equidade revelou uma lacuna crucial: embora as intervenções considerem determinantes sociais da saúde, a maioria das revisões sistemáticas falhou em analisar o efeito diferenciado desses fatores nos resultados. 

Este achado sugere que fatores como raça, gênero, status socioeconômico e capital social afetam a eficácia das intervenções e podem ampliar as iniquidades. Assim, esta síntese aponta para a necessidade urgente de futuras pesquisas e políticas públicas considerarem e analisarem ativamente esses determinantes para garantir intervenções mais inclusivas e sensíveis às desigualdades sociais.

## Data Availability

Todos os dados utilizados para este overview, incluindo a matriz de extração e a avaliação de qualidade dos estudos, estão disponíveis por meio do contato com o autor correspondente: angelo.costa@pucrs.br

## Tabela Suplementar

Tabela Suplementar 1
